# Unravelling the genomic features, phylogeny and genetic basis of tooth ontogenesis in Characiformes through analysis of four genomes

**DOI:** 10.1093/dnares/dsad022

**Published:** 2023-10-03

**Authors:** Xianwei Yang, Yue Song, Rui Zhang, Mengjun Yu, Xinyu Guo, Haobing Guo, Xiao Du, Shuai Sun, Chunhua Li, Xuebin Mao, Guangyi Fan, Xin Liu

**Affiliations:** College of Life Sciences, University of Chinese Academy of Sciences, Beijing 100049, China; BGI Research, Qingdao 266555, China; BGI Research, Qingdao 266555, China; BGI Research, Qingdao 266555, China; BGI Research, Qingdao 266555, China; BGI Research, Qingdao 266555, China; BGI Research, Qingdao 266555, China; BGI Research, Shenzhen 518083, China; College of Life Sciences, University of Chinese Academy of Sciences, Beijing 100049, China; BGI Research, Qingdao 266555, China; BGI Research, Qingdao 266555, China; BGI Research, Qingdao 266555, China; BGI Research, Qingdao 266555, China; BGI Research, Shenzhen 518083, China; College of Life Sciences, University of Chinese Academy of Sciences, Beijing 100049, China; BGI Research, Shenzhen 518083, China

**Keywords:** Characiformes, whole-genome sequencing, phylogenetic analysis, tooth ontogenesis

## Abstract

Characiformes is a diverse and evolutionarily significant order of freshwater fish encompassing over 2,300 species. Despite its diversity, our understanding of Characiformes’ evolutionary relationships and adaptive mechanisms is limited due to insufficient genome sequences. In this study, we sequenced and assembled the genomes of four Characiformes species, three of which were chromosome-level assemblies. Our analyses revealed dynamic changes in gene family evolution, repeat sequences and variations in chromosomal collinearity within these genomes. With the assembled genomes, we were not only able to elucidate the evolutionary relationship of the four main orders in Otophysi but also indicated Characiformes as the paraphyletic group. Comparative genomic analysis with other available fish genomes shed light on the evolution of genes related to tooth development in Characiformes. Notably, variations in the copy number of secretory calcium-binding phosphoproteins (SCPP) genes were observed among different orders of Otophysi, indicating their potential contribution to the diversity of tooth types. Our study offers invaluable genome sequences and novel insights into Characiformes’ evolution, paving the way for further genomic and evolutionary research in fish.

## 1. Introduction

The order of Characiformes belongs to Otophysi clade, which is a sub-group of the superorder Ostariophysi. Ostariophysi, a tremendously diversified group of teleost fish,^1,2^ accounting for about 68% of the world’s freshwater fish and consists of Otophysi and Anotophysi,^3^ of which the former has four orders including Cypriniformes, Siluriformes, Characiformes and Gymnotiformes, while the latter has only one order Gonorynchiformes. Previous morphological and molecular studies proposed three major hypotheses on the phylogenetic relationship within Otophysi,^3–8^ making it a long-standing issue in fish evolution. Within Otophysi, Characiformes contains 24 recognized families with over 2,300 species.^1,9^ They play a crucial role in Neotropical freshwater ecosystems and exhibit significant morphological diversity. Many members of this group have a body length of less than 3 cm, while the largest member, *Hydrocynus goliath*, can reach a length of up to 1.4 m.^1^ The dietary habits of Characiformes fish are also diverse, as they typically occupy various ecological niches within different ecosystems. Some are carnivorous predators, while others are herbivores, and some even dwell in benthic environments. This diversity underscores the importance of Characiformes as a subject for ecological. Despite the significant species diversity in Characiformes, only six species from four families have been sequenced with whole genome assembly, of which only three genome assemblies were in the chromosome level. As a result, the classification of Characiformes into two suborders, Characoidei and Citharinoidei,^1^ raises the question of whether Characiformes is monophyletic or paraphyletic. With more genomes to be sequenced and assembled in Otophysi, especially whole genomes of species from the under-representative Characiformes order, it would be feasible to resolve these evolutionary controversies.

Mineralization plays a critical role in vertebrate evolution. As a mineralized organ composed of enamel, dentine, cementum and supported by the surrounding alveolar bone, the tooth serves as an adaptive characteristic that co-varies with nutrition and dietary habits.^10^ Previous studies have revealed that a number of proteins, including secretory calcium-binding phosphoprotein (SCPP), integrin subunit alpha V (ITGAV, also known as CD51) and kringle containing transmembrane protein (KREMEN), are closely involved in tooth biomineralization and differentiation.^11–17^ Ray-finned fish, which constitute a prominent subgroup of vertebrates, display a diverse range of tooth morphology in terms of tooth number, shape, size and localization,^18,19^ making them excellent models for studying the tooth differentiation in non-mammalian vertebrates.^20,21^ Dental patterns and tooth types vary significantly within ray-finned fish, even among clades such as Cypriniformes, Characiformes and Siluriformes, which all belong to Otophysi. For instance, all Cypriniformes species lack oral and anterior pharyngeal teeth, with the remaining teeth found only on the ventral fifth ceratobranchial bones.^19,22^ Siluriformes species, commonly known as catfish, are omnivorous and typically exhibit patches of small conical teeth in their mouth.^23,24^ Characiformes species, despite having diverse food sources including plants, insects or other fish,^1^ commonly exhibit well-developed and multi-cuspid jaw teeth. These teeth can be found on marginal bones of the oral jaws or even outside the mouth.^25–27^ Given their typical and unique jaw tooth types, Characiformes can be used to study the genetic basis of tooth specificity and development with genome sequences available.

In this study, we selected four representative species from Characiformes, including *Acestrorhynchus altus* (ITIS TSN: 639855), *Hepsetus odoe* (ITIS TSN: 163071), *Semaprochilodus insignis* (ITIS TSN: 641718) and *Distichodus sexfasciatus* (ITIS TSN: 163212). These four species exhibit variations in body size and feeding habits and they belong to different families, namely Acestrorhynchidae, Hepsetidae, Prochilodontidae and Distichodontidae, respectively. The first three species belong to the suborder Characoidei, while *D. sexfasciatus* belongs to the suborder Citharinoidei. We sequenced and assembled the genomes of these four Characiformes species and analysed them to explore their genome features, evolutionary relationships, as well as possible genetic mechanisms underlying Characiformes’ tooth ontogenesis.

## 2. Materials and methods

### 2.1. Sample collection and sequencing

All the samples were purchased from YueHe Flower-Bird-Fish Market in Shenzhen, Guangdong Province, China. Photos were taken to document their status (Supplementary Fig. S1). Muscles from each fish sample were obtained for further genomic DNA and RNA extraction. For whole genome sequencing, we referred to the published protocols for DNA and RNA extraction.^28^ We only successfully obtained RNA from *Acestrorhynchus altus*; thus, its RNA was subjected to further library construction and sequencing. Single-tube long fragment read (stLFR) libraries were constructed using the extracted DNA according to the previously published procedure.^29^ The *in situ* Hi-C library was constructed from the obtained muscle tissues of three species (*A. altus*, *S. insignis* and *H. odoe*) following the previous protocol^30^ with some modifications. We used the restriction endonuclease MboI for DNA digestion, followed by biotinylated residue labelling. All the stLFR, RNA and Hi-C library were sequenced on BGISEQ-500 platform. Finally, for the three species with enough long fragment DNA extracted (*A. altus*, *H. odoe* and *D. sexfasciatus*), we used Nanopore GridION to sequence long reads, according to the manufacturer’s instructions.

### 2.2. Genome assembly and annotation

With the stLFR sequencing data, we first used SOAPnuke (v1.6.5)^31^ to filter reads of low quality (i.e. >40% low-quality bases, *Q* < 7), reads resulted from PCR duplication and reads affected by adapter contamination. Then, the barcode sequences were extracted with the stLFR tool available at GitHub (https://github.com/stLFR/stLFR_read_demux). Based on filtered stLFR reads, we used Jellyfish (v2.2.6)^32^ and Genomescope (v1)^33^ for kmer frequency calculation (setting k to 17) and the genome size estimation. The stLFR data were then subjected to genome assembly using the pipeline stLFR2supernova (https://github.com/BGI-Qingdao/stlfr2supernova_pipeline), which implemented Supernova assembler (v2.0.1, https://www.10xgenomics.com/) to assemble stLFR reads. Inner gaps in the assembled sequences were filled in by GapCloser (v1.2)^34^ and then further gap filling was fulfilled by TGS-GapFiller^35^ using Nanopore data for the three species with Nanopore data available (*A. altus*, *H. odoe* and *D. sexfasciatus*), resulting in intermediate genome assemblies. The Hi-C data were processed using HiC-Pro pipeline (v2.8.0)^36^ with default parameters, and then Juicer (v.1.5)^37^ was used for alignment against the respective intermediate genome assemblies. Finally, we applied 3D-DNA workflow (3D *de novo* assembly, v170123)^38^ to create the ordered-and-oriented genome sequences in chromosome level with the main parameters setting as ‘-m haploid -s 4’, which resulted in the final genome assemblies (for *A. altus*, *S. insignis* and *H. odoe*). Since we were not able to obtain qualified Hi-C data for *D. sexfasciatus*, we only obtained scaffold level genome assembly for *D. sexfasciatus* as the final result. For each of the four sequenced species, we randomly extracted 10 million paired-reads from the clean stLFR data to assemble the mitochondrial genome using MitoZ (v2.3).^39^

To facilitate gene prediction in the assembled genomes, we first annotated the repetitive elements. We applied both the *de novo* and the homology-based repeat annotation pipelines. In the *de novo* annotation, we constructed a *de novo* repeat library using RepeatModeler (v1.0.8)^40^ and LTR-FINDER (v1.0.6)^41^ and then predicted the interspersed repetitive elements by RepeatMasker (v4.0.5).^42^ In the homology-based annotation, we detected interspersed repeats by aligning the genomes against the Repbase database at DNA and protein levels using RepeatMasker and RepeatProteinMask (v4.0.5).^42^ Tandem repeats were predicted using TRF (v4.09),^43^ a tool for identifying tandem repeat sequences in the genome.

We then conducted structural and functional gene annotation for the assembled genomes. In gene structural annotation, we used both homology-based and *de novo* pipelines. In *de novo* annotation, we used AUGUSTUS (v3.1)^44^ to predict gene models with zebrafish genes as the training dataset. We carried homology-based annotation based on proteins from eight-related species including *Astyanax mexicanus*, *Cyprinus carpio*, *Danio rerio*, *Gadus morhua*, *Gasterosteus aculeatus*, *Oryzias latipes*, *Pygocentrus nattereri* and *Takifugu rubripes*. GeneWise (v2.4.1)^45^ was used to predict gene models by first aligning these protein sequences against the genome and then determine the gene structures. For the species with RNA sequencing data (*A. altus*), we also assembled the transcripts using TRINITY (v2.8.5)^46^ based on the clean data after filtering by SOAPnuke (v1.6.5)^31^ with the parameters ‘-M 1 -A 0.4 -n 0.05 -l 10 -q 0.4 -Q 2 -G -5 0’. We further apply HISAT2 (v2.1.0),^47^ StringTie (v1.3.4)^48^ and PASA (v2.3.3)-TransDecoder^49^ to predict the gene models based on the assembled transcripts. Finally, we used GLEAN (http://sourceforge.net/projects/glean-gene)^50^ to integrate gene models predicted by all the above mentioned pipelines and obtain a final non-redundant gene set. The functional gene function was fulfilled by aligning the predicted genes against TrEMBL,^51^ Swissprot,^51^ InterPro,^52^ Gene Ontology^53^ and Kyoto Encyclopedia of Genes and Genomes (KEGG).^54^ The overall repeat, gene and gene function annotation process was illustrated in Supplementary Fig. S2.

### 2.3. Genome alignment and phylogenetic analysis

We obtained protein sequences of nine Otophysi species (*A. altus*, *Astyanax mexicanus*, *H. odoe*, *Pygocentrus nattereri*, *S. insignis*, *D. sexfasciatus*, *Ictalurus punctatus*, *Electrophorus electricus* and *D. rerio*) and one outgroup species (*Lepisosteus oculatus*). Other than the genes we annotated in four species sequenced in this study, the protein-coding genes of the other six species were downloaded from NCBI. We also removed genes with frameshift, shorter than 50 amino acids, and the redundant copies, only to remain the longest transcripts for comparative genomic analysis. All-versus-all protein similarities were computed by BLASTP^55^ and the alignment results were used through TreeFam (v4.0)^56^ to deduce homologous gene sequences and identify gene families. Orthologue clustering analysis of predicted genes was conducted using MCL algorithm.^57^ We also applied cactus (v.2.0.5)^58^ to perform whole genome alignments with default parameters.

To resolve the phylogenetic relationships and divergence times, we constructed phylogenetic trees using single-copy genes shared by all species, as well as the genome-wide aligned sequences. The amino acid sequences of shared single-copy genes were aligned by MUSCLE (v3.8.31)^59^ with default parameters. For the genome-wide aligned sequences, we concatenated the aligned sequences into a super alignment sequence for each species. These alignments were subjected to RAxML (v8.2.4),^60,61^ separately, for phylogeny construction. In RAxML, we used a random number of ‘12345’ in each independent Maximum Likelihood (ML) tree search with the best amino acid substitution model JTT+Γ and nucleotide substitution model GTR+Γ applied. Clade supports were assessed using 1,000 non-parametric bootstrap replicates. Divergence time was inferred using the MCMCTree in PAML package (v4.7a).^62^ The fossil time between *E. electricus* and *I. punctatus* (108.1–148.2 million years ago, Mya), and between *P. nattereri* and *D. rerio* (132–170 Mya) was obtained from TimeTree database (http://www.timetree.org/) and used for time recalibration.

We also carried out pairwise comparisons between species with chromosome-level genomes at gene level with BLASTn setting E-value to be 10e-5. Then, we used MCScanX,^63^ a software program that detects synteny blocks, to identify chromosomal rearrangements.

### 2.4. Analysis on the tooth related genes

To depict the evolution and diversification of tooth-related genes in fish, we first collected 83 genes from previous publications and established a tooth-related gene dataset with KO (KEGG Orthology) entry (Supplementary Table S1). Given that the sequence of the same gene varies in different species, we searched these genes from the KEGG database (https://www.kegg.jp/kegg/) by KO id and obtained the amino acid sequences that appeared in 13 representative species (*Homo sapiens*, *Mus musculus*, *Gallus gallus*, *Chelonia mydas*, *Xenopus laevis*, *Danio rerio*, *Takifugu rubripes*, *Oryzias latipes*, *Cynoglossus semilaevis*, *Anguilla anguilla*, *Lepisosteus oculatus*, *Latimeria chalumnae* and *Callorhinchus milii*). Given that the secretory calcium-binding phosphoproteins (SCPP) family in the KEGG database is incomplete and suffered rapid evolution, we sought to incorporate additional sources of data. This included 38 SCPP genes identified in gars (*L. oculatus*).^17^ We further supplemented our dataset with 4,004 protein sequences obtained from the NCBI database. Finally, we obtained 6,119 amino acid sequences as the query dataset. We used blat,^64^ to search the query sequences against 69 genomes (including the ones we assembled and downloaded online), which covering 2 orders from Chondrichthyes and 47 orders from Osteichthyes. In order to avoid the interference by non-coding regions, we extended the alignment to 1,000 bp upstream and 1,000 bp downstream flanking regions, and used GeneWise (v2.4.1)^45^ to predict the gene structure based on the alignments. For the SCPP gene, some sequences contained regions of low complexity, which may result in unreliable gene predictions. We employed blastp (blastall v2.2.26)^55^ to mask low-complexity amino acid sequences. Finally, We Finally, we filtered out all identified genes with an amino acid length shorter than 30, to filter incomplete genes.

## 3. Results

### 3.1. Genome sequencing and assembly

For the four species *Acestrorhynchus altus*, *Hepsetus odoe*, *Semaprochilodus insignis* and *Distichodus sexfasciatus*, we obtained 125.5 Gb (~132X), 118.6 Gb (~137X), 120.7 Gb (~102X) and 125.1 Gb (~154X) stLFR data, respectively (Supplementary Table S2). These data allowed us to generate preliminary scaffold level genome assemblies. To further improve the continuity of genome assemblies, we produced 11.3 Gb, 11.0 Gb and 12.2 Gb Nanopore long reads for *A. altus*, *H. odoe* and *D. sexfasciatus*, respectively. This refinement process notably increased the contig N50 from 15 kb to 556 kb, from 23 kb to 352 kb and from 32 kb to 414 kb, respectively. To anchor scaffolds in the preliminary assemblies or improved preliminary assemblies to chromosomes, we generated 145 Gb, 67 Gb and 77 Gb Hi-C data for *A. altus*, *H. odoe* and *S. insignis*, respectively. This allows us to anchor 88.7%, 88.5% and 78.0% of scaffolds onto 25, 29 and 27 chromosomes, respectively (Supplementary Fig. S3; Supplementary Table S3). The final genome assemblies were 952.8 Mb for *A. altus*, 864.4 Mb for *H. odoe*, 1.18 Gb for *S. insignis* and 813.5 Mb for *D. sexfasciatus* (Table 1), representing ~96%, ~93%, ~79% and 100% of the estimated genome sizes (Supplementary Fig. S4).

**Table 1.** Statistics of the genome assemblies.

	*Acestrorhynchus altus* (CNA0051955)	*Hepsetus odoe* (CNA0051954)	*Semaprochilodus insignis* (CNA0051956)	*Distichodus sexfasciatus* (CNA0051957)
Total sequence length	952,761,742	864,433,607	1,179,369,281	813,537,017
Total ungapped length	940,913,899	848,188,718	1,075,976,666	800,902,929
Number of scaffolds	19,140	25,484	65,578	36,008
Scaffold N50	34,115,393	27,055,951	32,752,913	1,041,799
Scaffold L50	12	15	15	147
Number of contigs	24,116	31,764	116,955	38,672
Contig N50	555,654	352,135	29,766	414,085
Contig L50	505	680	8,613	479
Genome Complete BUSCOs (C)	4,349 (94.9%)	4,330 (94.5%)	3,826 (83.5%)	4,146 (90.4%)
Genome Complete and single-copy BUSCOs (S)	4,064 (88.7%)	4,117 (89.8%)	3,649 (79.6%)	3,919 (85.5%)
Gene Complete BUSCOs (C)	4,191 (91.4%)	4,237 (92.4%)	3,703 (80.8%)	4,046 (88.3%)
Gene Complete and single-copy BUSCOs (S)	3,847 (83.9%)	3,947 (86.1%)	3,449 (75.2%)	3,780 (82.5%)

In addition to the completeness comparing to estimated genome size, the assembled genomes were of high quality, reflected by continuity, as well as genome and gene region completeness estimated by Benchmarking Universal Single-Copy Orthologs. Moreover, for *A. altus*, *H. odoe* and *S. insignis*, we anchored genome sequences to chromosomes, adding chromosome level assemblies for Characiformes and making the chromosomal evolutionary analysis feasible. With the whole genome sequencing data, we also assembled the complete mitochondrial genomes of these four species, which were rotated to a circular sequence with total length of 16,839 bp, 16,692 bp, 16,765 bp and 16,555 bp in *A. altus*, *H. odoe*, *S. insignis* and *D. sexfasciatus*, respectively (Supplementary Fig. S5).

**Figure 1.**
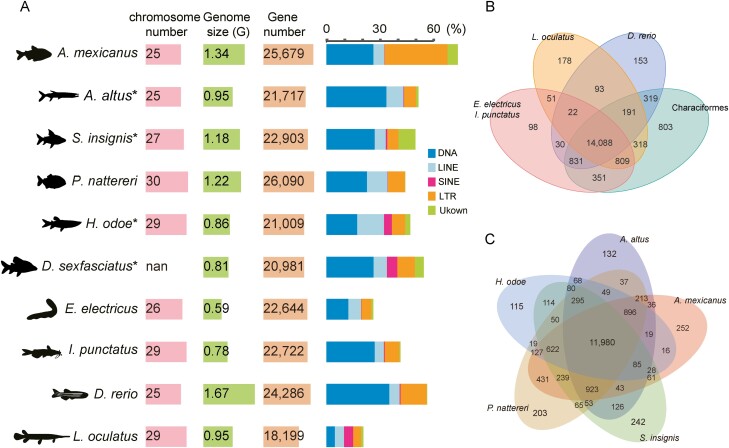
The assembled genomes. (A) Bar graph of genome size, number of genes and proportion of repeat regions. Meanwhile, the assembled chromosome number of each genome was marked. (B) Venn diagram of gene families among six organisms from Characiformes and four other species. (C) The shared gene families among five Characoidei species was depicted by the Venn diagram.

With the genome sequences assembled, we then annotated the genomes to obtain genomic features including repeat content and gene content. We found the average repeat content to be 39.5% in these four genomes, ranging from 34.5% in *H. odoe* to 44.4% in *A. altus* (Table 2). We found the DNA repeats to be the most abundant repeats for all the four genomes, accounting for a high percentage of the total repeats. In addition to DNA repeats, either long interspersed nuclear elements or long terminal repeats (LTRs) were the second abundant repeats, and notably, we found substantial amounts of repeats to be annotated as unknown, which might indicate specific repeats in Characiformes or possible discrepancies in the repeat annotation method. Furthermore, we found the high proportion of LTRs (35.4%, comparing to 26.5% DNA repeats) in *Astyanax mexicanus*, should have contributed to its relatively large genome (1.29 Gb), which might result from specific LTR bursts after its speciation with *A. altus* (with 6.7% LTRs). Overall, we found the Characiformes genomes to be abundant in DNA repeats, with some repeat families to be changed in specific subgroups, resulting in varied repeat contents (Fig. 1A).

**Table 2.** Repeat content and protein coding genes in the assembled genomes and related genomes.

		DNA	LINE	SINE	LTR	Others	Unknown	Total repeat	Gene number
*Astyanax mexicanus*	Length (bp)	353,512,506	81,845,031	2,158,484	472,077,722	2,549	76,769,586	779,532,887	25,679
	% of whole genome	26.5	6.1	0.2	35.4	0.0	5.8	58.4	
Acestrorhynchus altus*	Length (bp)	321,871,278	91,608,661	3,322,747	64,069,010	72,234	12,113,547	422,749,596	21,717
	% of whole genome	33.8	9.6	0.3	6.7	0.0	1.3	44.4	
Semaprochilodus insignis*	Length (bp)	321,033,373	73,257,687	8,987,532	75,344,559	719	111,775,434	472,500,614	22,903
	% of whole genome	27.2	6.2	0.8	6.4	0.0	9.5	40.1	
*Pygocentrus nattereri*	Length (bp)	279,041,933	141,293,418	2,606,261	117,363,782	558	1,340,623	464,738,559	26,090
	% of whole genome	22.8	11.6	0.2	9.6	0.0	0.1	38.0	
Hepsetus odoe*	Length (bp)	149,857,298	130,106,409	38,578,602	65,195,162	2,535	24,166,658	297,930,653	21,009
	% of whole genome	17.3	15.1	4.5	7.5	0.0	2.8	34.5	
Distichodus sexfasciatus*	Length (bp)	216,959,267	61,022,326	47,490,309	80,418,104	37,849	40,217,855	316,906,493	20,981
	% of whole genome	26.7	7.5	5.8	9.9	0.0	4.9	39.0	
*Electrophorus electricus*	Length (bp)	72,765,712	42,049,933	1,439,583	32,945,851	746	5,958,443	125,448,436	22,644
	% of whole genome	12.3	7.1	0.2	5.6	0.0	1.0	21.3	
*Ictalurus punctatus*	Length (bp)	212,716,276	40,400,130	4,016,783	63,863,908	3,072	5,415,046	285,434,830	22,722
	% of whole genome	27.2	5.2	0.5	8.2	0.0	0.7	36.4	
*Danio rerio*	Length (bp)	594,800,238	96,391,797	9,208,931	246,301,954	86	4,130,339	859,028,512	24,286
	% of whole genome	35.4	5.7	0.5	14.7	0.0	0.2	51.2	
*Lepisosteus oculatus*	Length (bp)	45,072,104	49,220,129	48,304,194	42,044,673	2,157	11,880,741	165,044,868	18,199
	% of whole genome	4.8	5.2	5.1	4.4	0.0	1.3	17.4	

^*^Those genomes newly obtained in this study were marked in white background.

We annotated ~20,000 protein coding genes in these four genomes (21,717 for *A. altus*, 21,009 for *H. odoe*, 22,903 for *S. insignis* and 20,981 for *D. sexfasciatus*), comparable to other closely related bony fishes. We identified 18,335 non-redundant gene families and 14,088 gene families were shared among *Electrophorus electricus*, *Ictalurus punctatus*, *Lepisosteus oculatus*, *Danio rerio* and Characiformes species, as well as 803 gene families to be specific in Characiformes (Fig. 1B). Furthermore, comparing within Characoidei, one of the suborders of Characiformes, we identified 11,980 shared gene families among 5 organisms and 132 families in *A. altus*, 115 families in *H. odoe*, 203 families in *Pygocentrus nattereri*, 242 families in *S. insignis* and 252 families in *Astyanax mexicanus* to be species-specific (Fig. 1C).

### 3.2. Reconstruction of the phylogenetic and chromosome evolution

**Figure 2.**
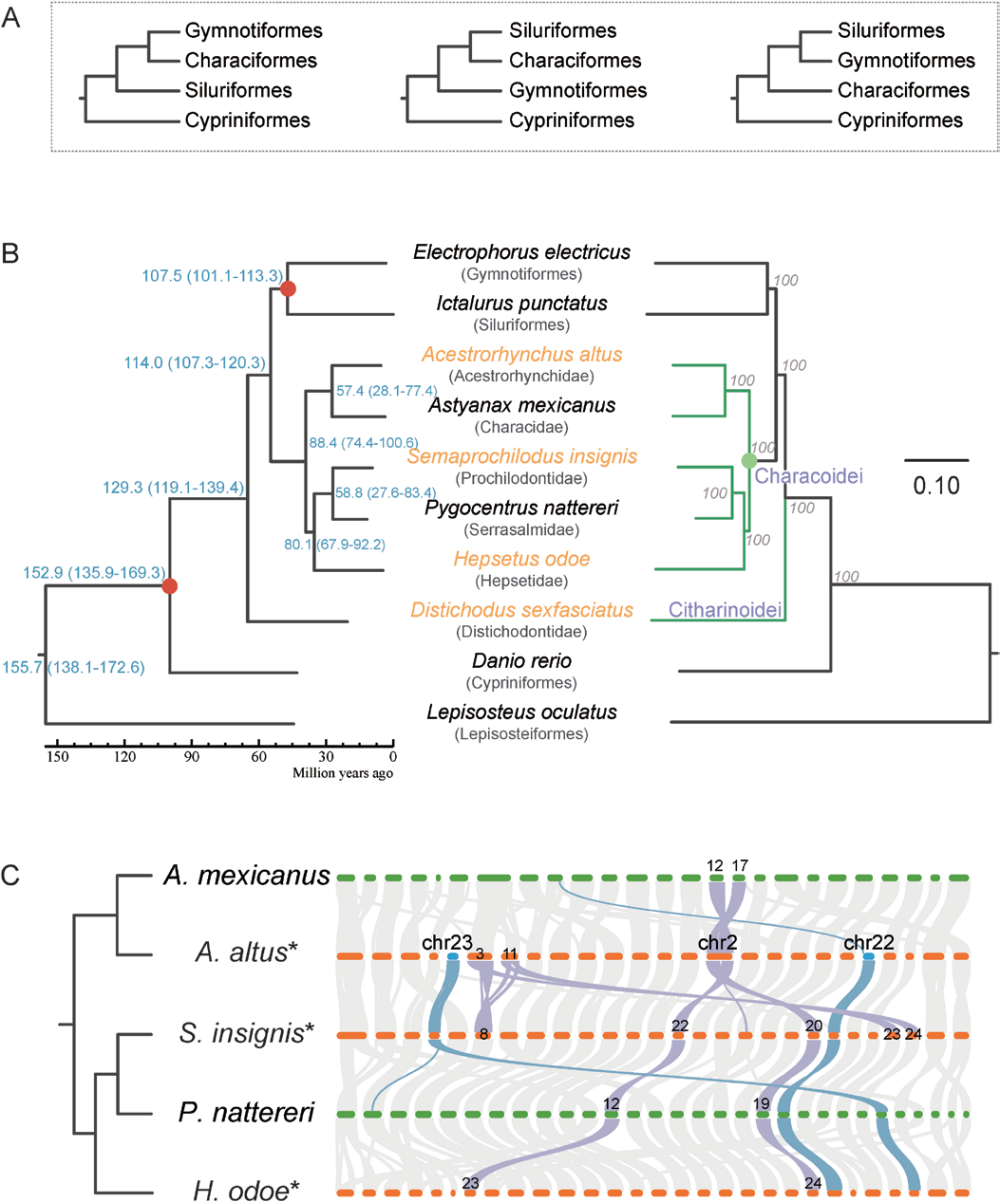
The phylogenetic relationship of Otophysi and chromosomal collinearity of Characiformes. (A) Three popular hypotheses about the phylogenetic relationship among four orders in Otophysi. (B) The phylogenetic relationship was rebuilt based on the dataset of single-copy genes (left) and whole genome alignments (right). In both trees, we carried out bootstraps validation with 1,000 and each branch achieved 100%. *Distichodus sexfasciatus*, which belongs to Characiformes:Citharinoidei, is independent of Characoidei. The genomes of species assembled in this study were colored orange. The time interval labelled on the node of branches in the left tree represents the estimated divergence time of the branches. Branches colored green in the right tree belong to Characiformes. (C) Collinear diagram between 5 Characiformes genomes, which at chromosome level, and the blocks associated with chromosome 2 and chromosome 22 in *A. altus* were highlighted.

Without enough whole genome sequences, the phylogenetic relationship among four orders in Otophysi was controversial with three different hypotheses (Fig. 2A).^5,6,8,65^ With the four genomes assembled in this study, as well as the six other genomes obtained from public databases, including *A. mexicanus* (GCF_000372685.2), *P. nattereri* (GCF_015220715.1), *D. rerio* (GCF_000002035.6), *E. electricus* (GCF_013358815.1), *I. punctatus* (GCF_001660625.1) and *L. oculatus* (GCF_000242695.1), we constructed the phylogenetic tree according to the whole genome sequences.

Single-copy genes can be particularly useful in evolutionary studies because their simpler inheritance patterns make them easier to track and analyse across different species, and they are often used to reconstruct phylogenetic relationships among species. For phylogeny construction, we identified 3,768 single-copy genes among 10 genomes. These genomes encompass 6 from Characiformes, 1 from Cypriniformes, 1 from Siluriformes, 1 from Gymnotiformes and *Lepisosteus oculatus*, which served as the outgroup. Meanwhile, we carried out whole genome alignment to obtain aligned sequences, also for phylogeny construction. We obtained 8,056 aligned blocks with a post-alignment length of 42,898,189bp. Thus, we obtained phylogenetic trees by setting *L. oculatus* as the outgroup, which are the genome wide information based phylogenetic tree for related species and phylogenetic groups for the first time, supported by multiple methods and with bootstrap values available.

Finally, we observed a consistency between the trees based on single-copy genes and whole genome alignment. The phylogenetic shows that *A. altus* and *A. mexicanus* were most closely related to *S. insignis*, *P. nattereri* and *H. odoe*, of which all belong to the suborder of Characoidei (Fig. 2B). The relationships within Characoidei were consistent with previous species classification.^66^ Meanwhile, the order of Siluriformes and Gymnotiformes, which were represented by *I. punctatus* and *E. electricus*, were more closely related to each other. Importantly, we found Characiformes to be the sister group to these two orders (Siluriformes and Gymnotiformes) and then to form a clade with Cypriniformes. Thus, our results supported the third hypothesis mentioned above, which was proposed through transcriptome analysis.^6^ Furthermore, within Characiformes, we found that Citharinoidei, one of the two suborders, to be located in an independent branch, supporting the Characiformes to be paraphyletic.

The karyotypic diversity in distinct families implies the pattern of chromosomal changes and related evolutionary processes.^67,68^ With the chromosomal genome assemblies available, we investigated chromosomal changes in Characiformes and related groups through synteny analysis. The chromosome numbers were different in the species used in phylogenetic analysis, ranging from 25 to 30.^69–72^ When comparing chromosomes of these species, we found a good chromosomal synteny between *A. altus* (2*n* = 50) and *S. insignis* (2*n* = 54), despite that they were from different phylogenetic clades. In total, we detected four major chromosomal differences between *A. altus* and *S. insignis*, related to three chromosomes in *A. altus* (Chromosome 2, 3 and 11) and five chromosomes in *S. insignis* (Chromosome 8, 20, 22, 23 and 24) (Fig. 2C). One of these differences indicated the collinearity between Chromosome 2 in *A. altus* and two chromosomes of *S. insignis* (Chromosome 20 and 22). Furthermore, Chromosome 2 of *A. altus* also had good collinearity with two chromosomes of *A. mexicanus* (Chromosome 17 and 12), and two chromosomes of *H. odoe* (Chromosome 24 and 23), indicating possible chromosome fusion event in *A. altus* resulting in its fewer chromosome number. Chromosomal fusion may disrupt existing genes or bring disparate genetic elements into close proximity.^73,74^ In the region of possible chromosome fusion on Chromosome 2 (25.5–28.7 Mb) of *A. altus*, we identified 30 novel genes comparing to *A. mexicanus* and *S. insignis*. These genes have functions related to metal ion binding, which might be introduced through the chromosomal rearrangements. Other than the possible chromosome fusion in *A. altus*, we also observed notable variations in gene orders of the *A. altus* and *A. mexicanus* genomes, despite that they have the same number of chromosomes and many conserved synteny blocks (Fig. 2C). Especially, we noted that the total sequence length of Chromosome 22 in *A. altus* is 23.76 M, with only 30 kb demonstrating synteny with Chromosome 2 of *A. mexicanus*. Moreover, Chromosome 23 of *A. altus*, which shows significant synteny with Chromosome 25 of *P. nattereri*, does not have an identifiable synteny block in *A. mexicanus* (Fig. 2C). The genes found on the Chromosome 22 and 23 of *A. altus* are scattered across multiple chromosomes in the genome of *A. mexicanus*. There are 560 and 447 genes on Chromosome 22 and 23, with 39 and 32 genes missing in *A. mexicanus*, respectively. Among these missing genes, 34 and 26 genes were identified as single-copy in *A. altus* (Table S4), which indicated gene dynamics resulted from chromosomal rearrangements. Altogether, we depicted notable chromosomal differences in Chraraciformes, and these chromosomal changes might be evolutionarily important with alterations in gene functions.

### 3.3. Genomic basis of tooth ontogenesis in Characiformes

Teeth are a crucial trait for vertebrate adaptation, and Characiformes species are characterized by well-developed and multicuspid teeth in their oral jaws. This is in contrast to closely related species from Cypriniforms (e.g. carps), which lack jaw teeth. Similarly, Siluriformes (e.g. catfishes) have a different dental pattern and typically possess patches of smaller, conical teeth in their mouth, displaying less complexity compared to the Characiformes species.^1^ Based on previous researches, we compiled a list of 83 genes or gene families that may be involved in tooth development (Supplementary Table S1). Using the protein sequences of these genes, we performed a homologous gene identification in Characiformes and their related species. In this way, we defined tooth ontogenesis genes in six species from Characiformes (including four sequenced and assembled in this study), four species from Cypriniforms and five species from Siluriformes (Supplementary Table S5). *S. insignis*, a species within the Prochilodontidae family, typically exhibits oral teeth that are notably smaller than those of other Characiforms. Our study revealed the lack of *KREMEN* and *ITGAV*/*CD51* genes in this species. These genes are recognized for their involvement in a variety of cellular processes that potentially have a direct influence on tooth development.^11,12^ We further analysed all the 83 tooth ontogenesis genes against 69 fish genomes (23 genomes of Otophysi and 46 genomes of non-Otophysi) from the Fish10K project,^75^ covering 49 orders of fish (Supplementary Fig. S6; Supplementary Table S6). Our analysis revealed that both *KREMEN* and *ITGAV/CD51* genes are prevalent in Actinopterygii, with a presence in 90% (61/68) and 81% (55/68) of the examined genomes, respectively. This discovery might provide some insight into the diminutive oral teeth size in *S. insignis* as a potential genomic correlate.

**Figure 3.**
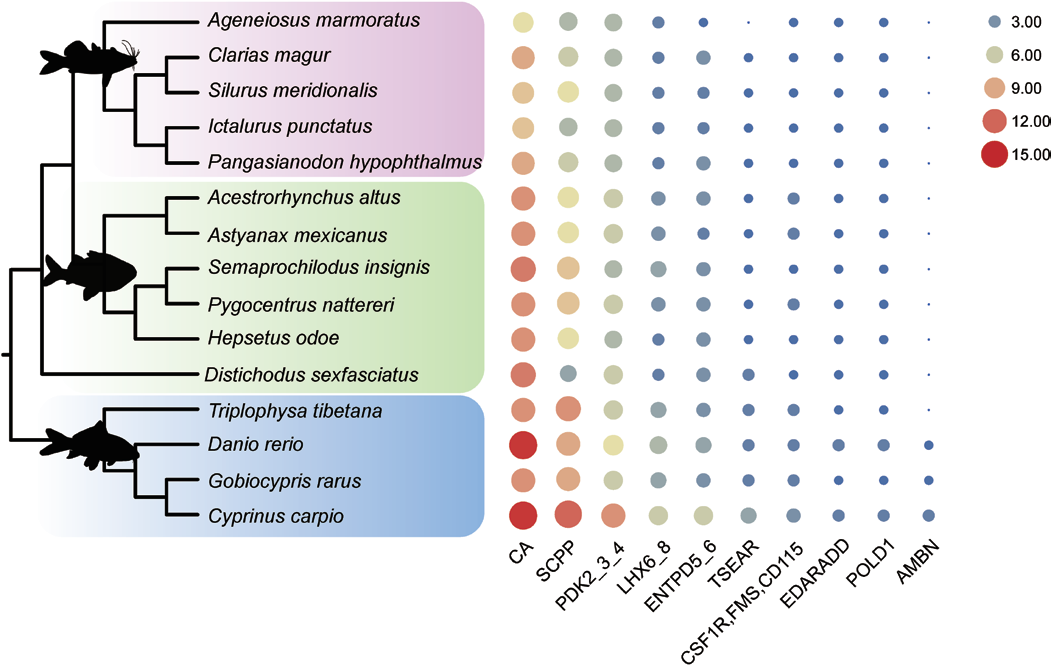
Gene copy number variation of tooth ontogenesis-related genes in fifteen species. The genomes used in this analysis included five Siluriformes (pink background), six Characiformes (green background) and four Cypriniformes (blue background). Bubble size indicates the copy numbers of 10 genes in each species.

Based on the copy number of these 83 genes, we found 10 genes with ANOVA test’s *P* value (intergroup comparison) lower than 0.05 (Table 3). We found that the copy number of secretory calcium-binding phosphoprotein (SCPP) gene was various among Ostariophysi (Fig. 3). This gene plays an essential role in tissue mineralization and tooth morphogenesis regulation.^76,77^ Consequently, the noticeable alterations in the copy number of this gene might be associated with the specific dental traits of Characiformes. Given that SCPP represents a superfamily encompassing numerous genes, we identified all *SCPP* genes in each species (Supplementary Fig. S7; Supplementary Table S7). Notably, the gene *scpp7*, which encodes P/Q-rich SCPPs and is notably expressed in the skin covered by ganoid scales and in the jaw of gar (*L. oculatus*),^17^ is absent in Siluriformes. However, this gene was identified within the genomes of Characoidei. This observation suggests intriguing divergence in the SCPP family across these different orders.

**Table 3.** Copy number of tooth ontogenesis related genes in 15 species.

Species	Order	CA^a^	SCPP	PDK2_3_4	LHX6_8	ENTPD5_6	TSEAR	CSF1R,FMS,CD115	EDARADD	POLD1	AMBN
*Ageneiosus marmoratus*	Siluriformes	7	5	5	2	1	0	1	1	1	0
*Clarias magur*	Siluriformes	9	6	5	2	3	1	1	1	1	0
*Silurus meridionalis*	Siluriformes	8	7	5	2	2	1	1	1	1	0
*Ictalurus punctatus*	Siluriformes	8	5	5	2	2	1	1	1	1	0
*Pangasianodon hypophthalmus*	Siluriformes	9	6	5	2	3	1	1	1	1	0
*Acestrorhynchus altus*	Characiformes	10	7	6	3	3	1	2	1	1	0
*Astyanax mexicanus*	Characiformes	10	7	6	3	2	1	2	1	1	0
*Semaprochilodus insignis*	Characiformes	11	8	5	4	3	1	1	1	1	0
*Pygocentrus nattereri*	Characiformes	10	8	6	3	3	1	2	1	1	0
*Hepsetus odoe*	Characiformes	10	7	5	2	3	1	1	1	1	0
*Distichodus sexfasciatus*	Characiformes	11	4	6	2	3	2	1	1	1	0
*Triplophysa tibetana*	Cypriniform	10	10	6	4	3	2	2	1	1	0
*Danio rerio*	Cypriniform	14	9	7	5	4	2	2	2	2	1
*Gobiocypris rarus*	Cypriniform	10	9	6	4	3	2	2	1	1	1
*Cyprinus carpio*	Cypriniform	14	12	10	6	6	4	3	2	2	2

CA: carbonic anhydrase (gene family); SCPP: secretory calcium-binding phosphoprotein (gene family); PDK2_3_4: pyruvate dehydrogenase kinase 2/3/4 (gene); LHX6_8: LIM homeobox protein 6/8 (gene); ENTPD5_6: ectonucleoside triphosphate diphosphohydrolase 5/6 (gene); TSEAR: thrombospondin-type laminin G domain and EAR repeat-containing protein (gene); CSF1R,FMS,CD115: macrophage colony-stimulating factor 1 receptor (gene); EDARADD: ectodysplasin-A receptor-associated adapter protein (gene); POLD1: DNA polymerase delta subunit 1 (gene); AMBN: ameloblastin (gene).

^a^Descriptions of genes/gene families.

## 4. Conclusion and discussion

We sequenced four new Characiformes genomes using different technologies, including the first genome of the Citharinoidei. By identifying single-copy genes and conducting whole genome alignments, we reconstructed the evolutionary relationships among Otophysi. Our results support hypothesis that Siluriformes and Gymnotiformes are more closely related while Characiformes are divided into two suborders belonging to different clades. Previous studies have implemented gene genealogy interrogation methodology to resolve controversial taxonomic groups,^78,79^ while in this study, we reconstructed the phylogenetic relationships of Otophysi using single-copy genes and genome-wide syntenic blocks. The phylogenetic relationships revealed using different methods were different, and it might be possible that our phylogenetic relationship was biased, with limited available genomes of Citharinoidei. Even though, these findings provide valuable insights into the evolution of Characiformes and Otophysi. Additional genomes can greatly enhance our understanding of the important fish groups, with improved the resolution of evolutionary relationships among species, which can help us to better understand the evolutionary history and diversification of these groups. Also, comparative analysis of more genomes can reveal genomic features associated with the evolution of specific traits, providing insights into the mechanisms of adaptation and the genetic basis of evolutionary change in Characiformes and Otophysi. Furthermore, new genes and genetic pathways important for the evolution and adaptation of these species can be discovered through additional genome sequencing, providing new targets for research in genetics, aquaculture, conservation, and biotechnology. Finally, comparison of Characiformes and Otophysi genomes with those of other fish groups can provide broader insights into the evolution of fish diversity, helping us to understand the patterns and processes of evolution across different fish groups and the factors that drive diversification and adaptation in aquatic environments.

Expanding previous findings of the association between genes and differences in tooth types, we further investigated the genetic basis of specialized jaw teeth in Characiformes by identifying homologous genes from 83 genes related to teeth development in 15 Otophysi organisms. Copy number variation of SCPP, particularly *scpp7*, which is absent in Siluriformes, and this may associated with their dental morphology. These findings enhance our understanding of specialized jaw teeth in Otophysi. Although we found these features of dental ontogenesis related genes to be possibly related with teeth features from sequence analysis, further validations on functions of these genes through experiments and future studies to elucidate the molecular mechanisms are required. Meanwhile, since tooth or dental ontogenesis is a complex process, involving multiple developmental stages and different biological metabolic processes, exploring different processes/stages/pathways, would be critical to clearly illustrate the mechanisms underlying dental ontogenesis.

## Supplementary Material

dsad022_suppl_Supplementary_Figures

dsad022_suppl_Supplementary_Legends

dsad022_suppl_Supplementary_Tables

## Data Availability

The amino acid sequence information for the previously mentioned genes, *KREMEN*, *ITGAV*, and *SCPP*, can be found in Supplementary Table S8. The genome assembly data for the four organisms in this study have been deposited into CNGB Sequence Archive (CNSA)^80^ of China National GeneBank DataBase (CNGBdb)^81^ with the accession number CNP0003776. Additionally, the data has also been deposited into the NCBI GenBank under the BioProject accession number PRJNA1004608.

## Statement of animal welfare

All experimental procedures involving animals were carried out in accordance with the guidelines set forth by the BGI Genomics Ethics Committee. All efforts were made to minimize animal suffering and to reduce the number of animals used.
