# Supplementary material for: Unravelling the genomic features, phylogeny and genetic basis of tooth ontogenesis in Characiformes through analysis of four genomes
Source: DNA Res. 2023 Oct 3;30(5):dsad022. doi: 10.1093/dnares/dsad022 (PMC10590162; doi:10.1093/dnares/dsad022)
Supplement: dsad022_suppl_Supplementary_Figures [file dsad022_suppl_supplementary_figures.pdf]

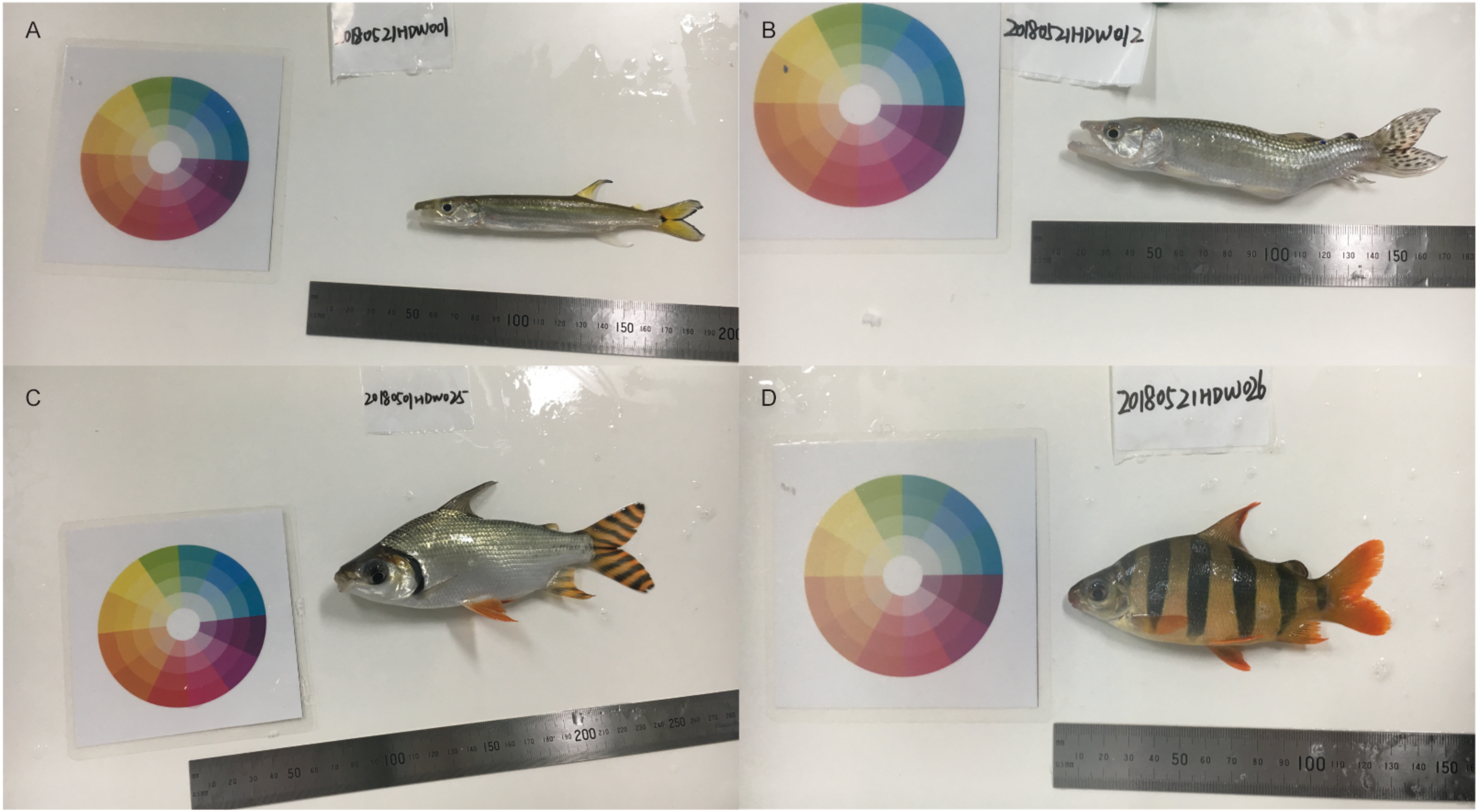

Supplementary Figure S1. The samples used in this study. *Acestrorhynchus altus* (A), *Hepsetus odoe* (B), *Semaprochilodus insignis* (C) and *Distichodus sexfasciatus* (D).

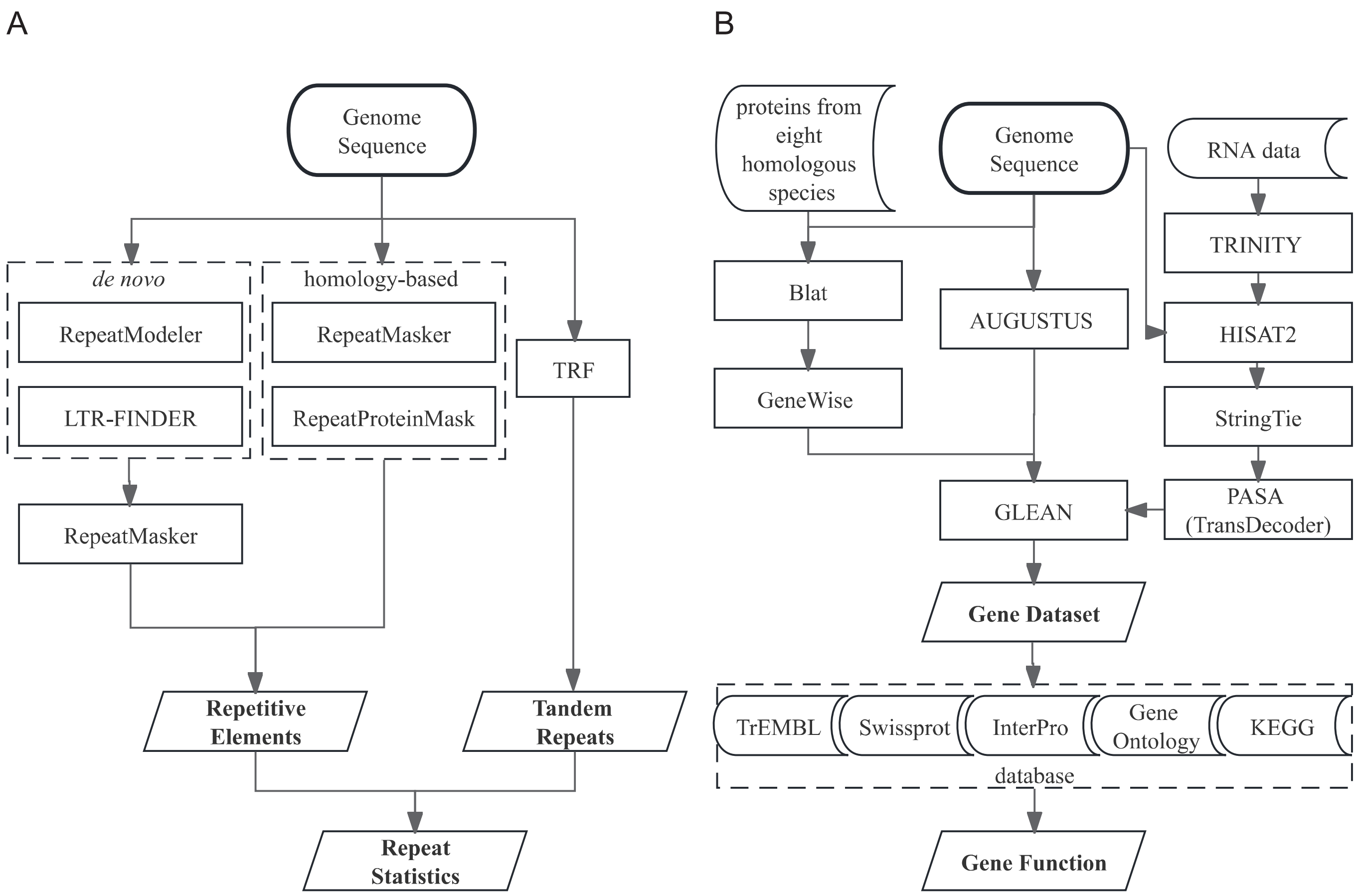

Supplementary Figure S2. The main pipeline and software used in whole genome repeat detection (A), and the gene identification and function prediction (B).

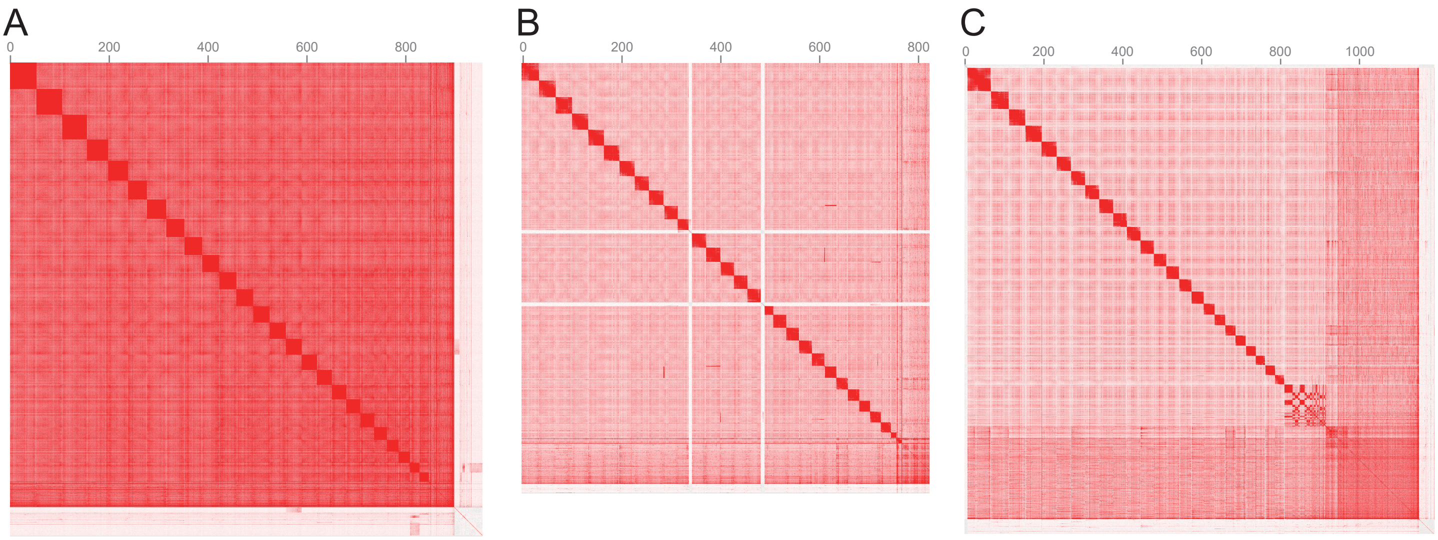

Supplementary Figure S3. Assembled to the chromosomal level with Hi-C data, the interaction heatmaps of *A. altus* (A), *H. odoe* (B) and *S. insignis* (C) with resolution of blocks at 500 kbp.

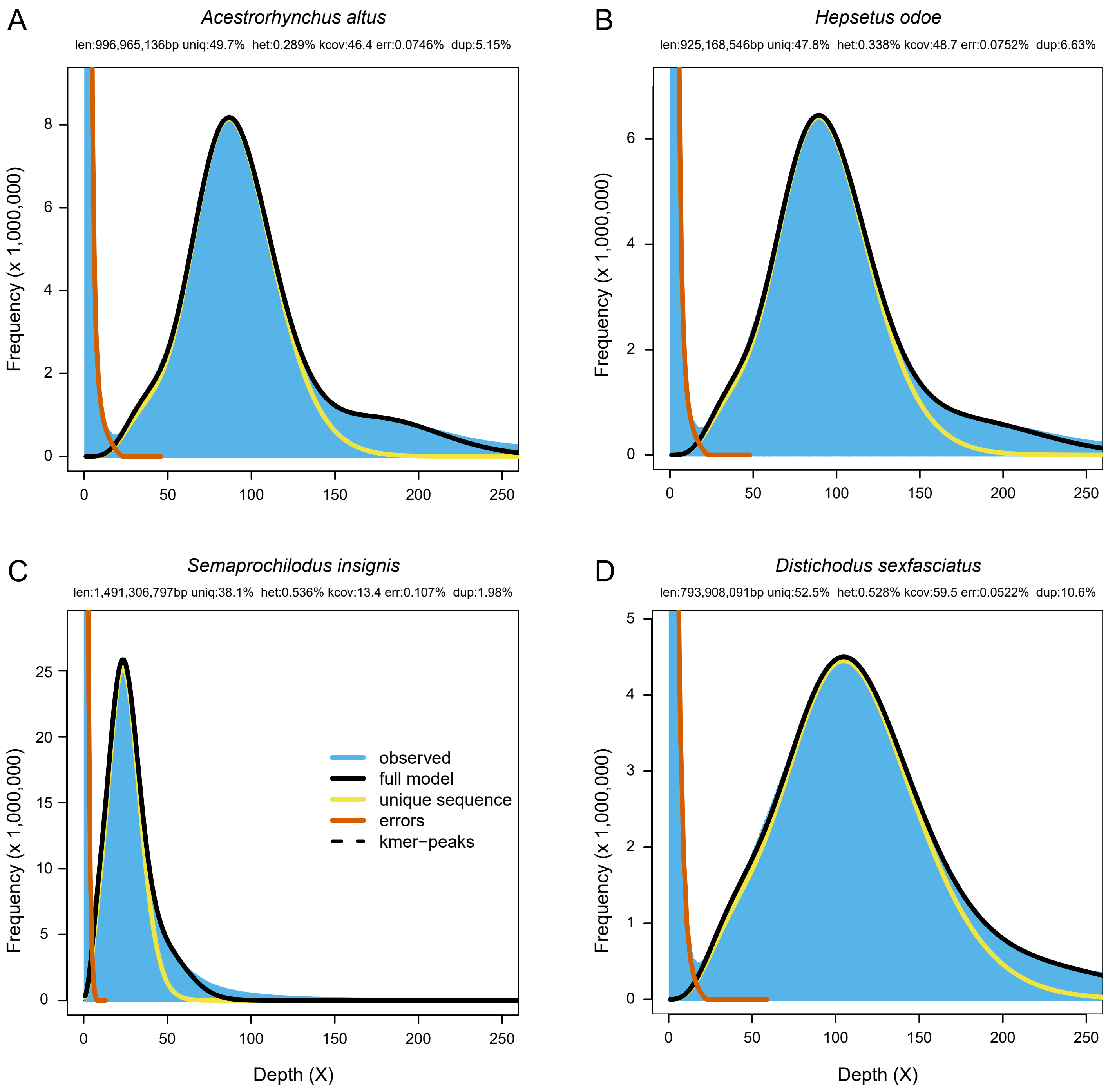

Supplementary Figure S4. The 17-mer frequency distribution of four genomes. The estimated genome size 997 M, 925 M, 793 M and 1.49 G separately.

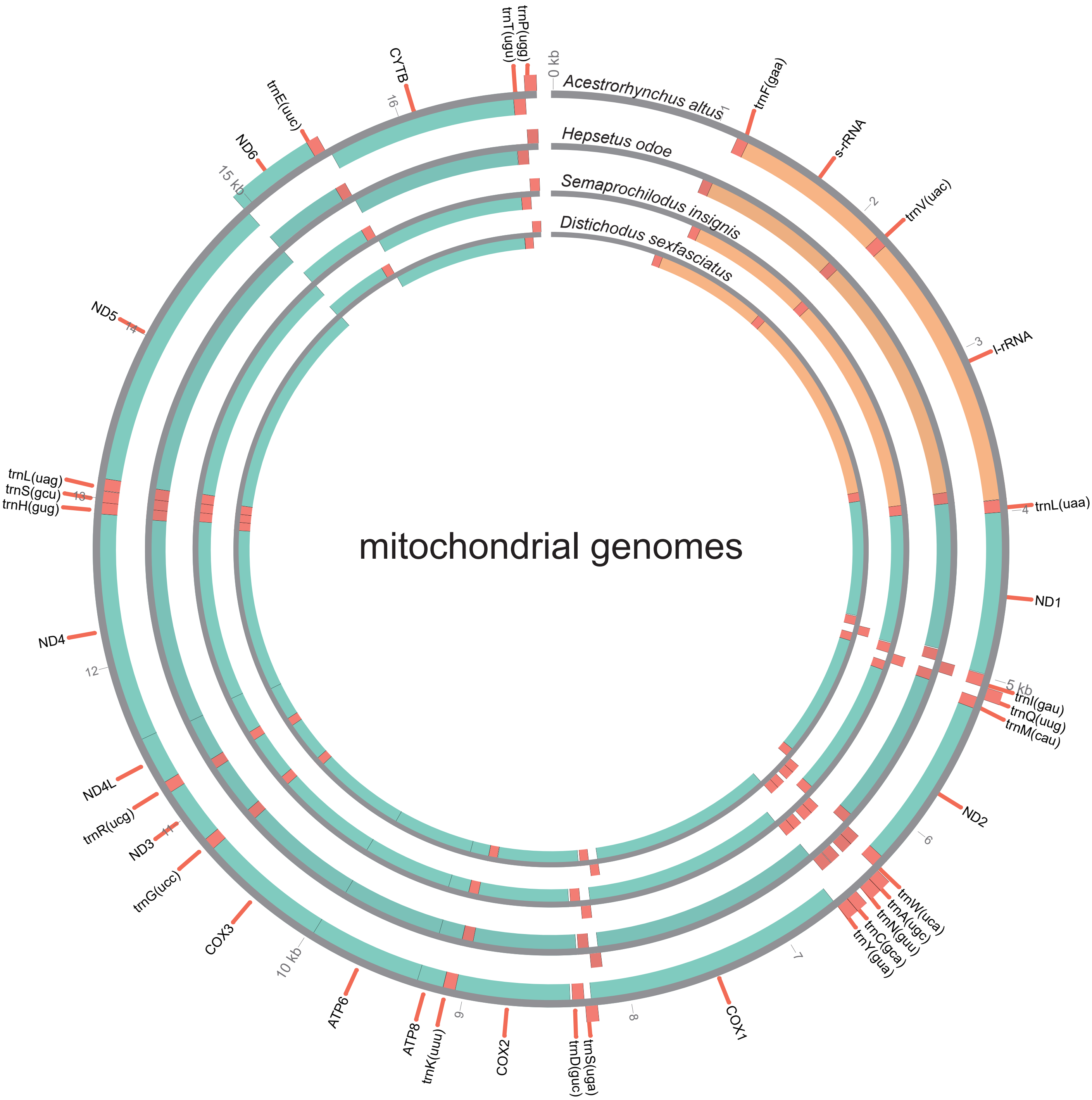

Supplementary Figure S5. Comparison and annotation of mitochondrial genomes, which belong to four organisms sequenced in this study.

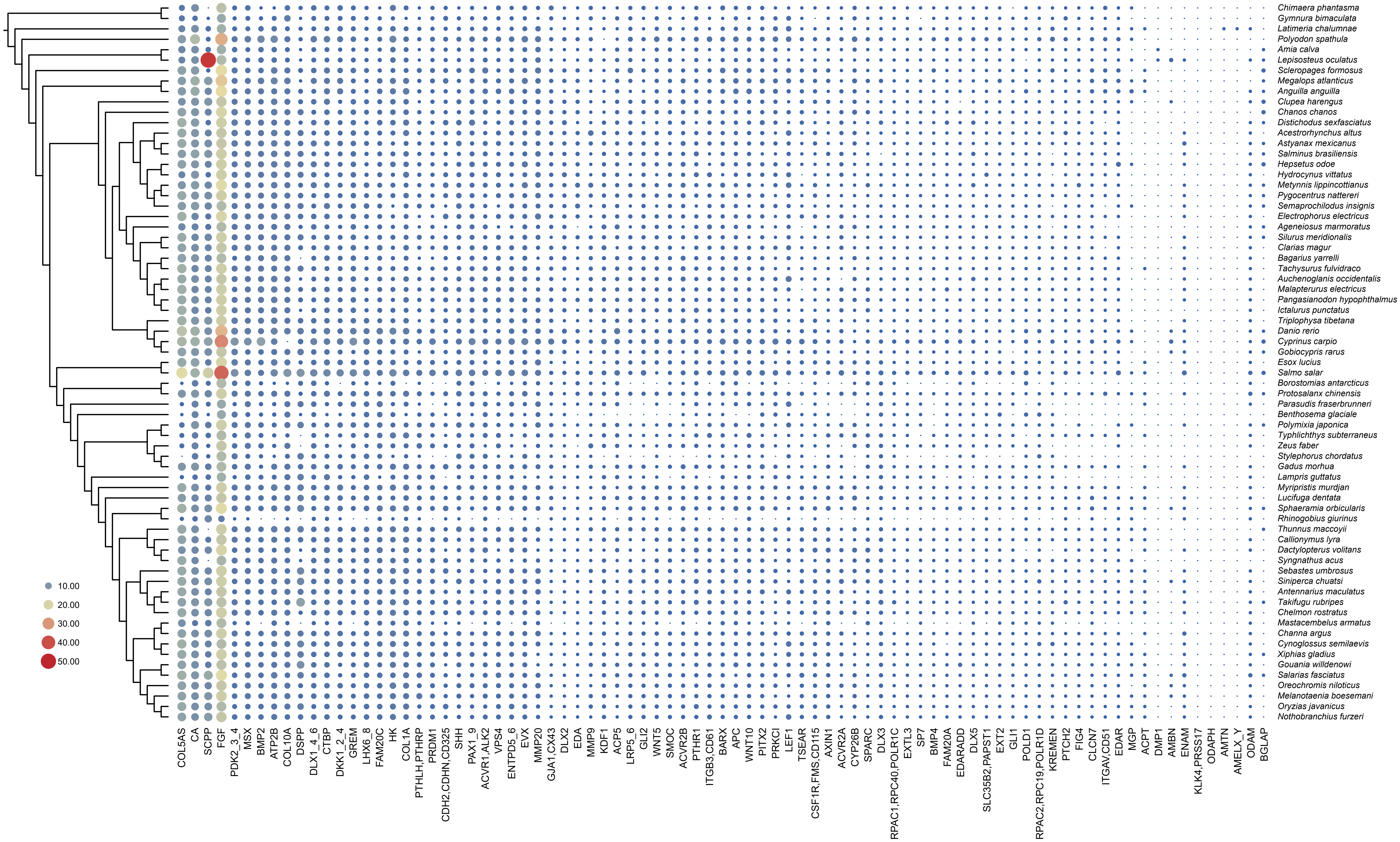

Supplementary Figure S6. The copy number of 83 genes in 69 species, which were sorted in taxonomy and phylogenetic relationships.

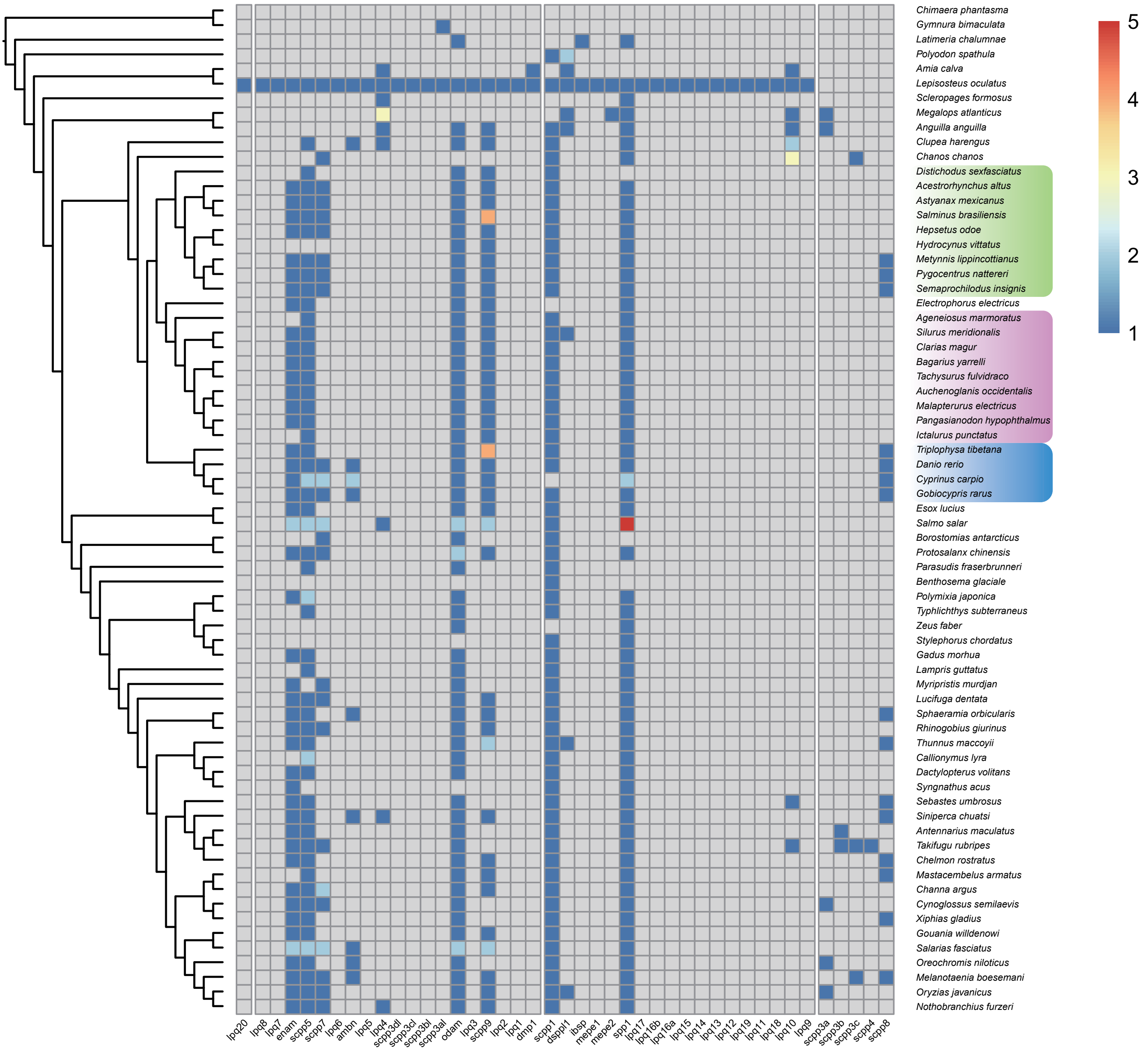

Supplementary Figure S7. The copy number of SCPP in 69 genomes. The previous 38 genes in the graph were sorted by the genome location in *L.oculatus*.
