# Supplementary material for: Unravelling the genomic features, phylogeny and genetic basis of tooth ontogenesis in Characiformes through analysis of four genomes
Source: DNA Res. 2023 Oct 3;30(5):dsad022. doi: 10.1093/dnares/dsad022 (PMC10590162; doi:10.1093/dnares/dsad022)
Supplement: dsad022_suppl_Supplementary_Legends [file dsad022_suppl_supplementary_legends.docx]

## **Supplementary data**

**Supplementary Table S1.** List of the tooth ontogenesis related genes. Genes with the same KEGG orthology id were merged and separated by comma in the first column. The symbol and function of genes were obtained from KEGG. The previous research about these genes was indicated by the DOIs. Despite multiple genes included in a gene family, the gene number used in this study were documented in the last column.

**Supplementary Table S2.** Summary of the sequencing data generated. The sequencing depth was calculated based on the assembled genome size.

**Supplementary Table S3.** Chromosomal assemblies by Hi-C data.

**Supplementary Table S4.** Table of gene comparisons from chromosomes 22 and 23 of *A. altus* versus *A. mexicanus*.

**Supplementary Table S5.** Copy number of tooth ontogenesis related genes in fifteen species.

**Supplementary Table S6.** Copy number of tooth ontogenesis related genes in 69 species.

**Supplementary Table S7.** The identified copy number of genes from the sub-families of SCPP. The gene names were ordered by the location in the genome of *L. oculatus*.

**Supplementary Table S8.** Predicted gene information for *KREMEN*, *ITGAV*, and SCPP. The table includes six columns: Gene symbol (include classification for SCPP gene family), Organism, Location (the genomic coordinates of the mRNA within the genome), Strand and Sequences (both the CDS and amino acid sequences).

**Supplementary Figure S1.** The samples used in this study. *Acestrorhynchus altus* (A), *Hepsetus odoe* (B), *Semaprochilodus insignis* (C) and *Distichodus sexfasciatus* (D).

**Supplementary Figure S2.** The main pipeline and software used in whole genome repeat detection (A), and the gene identification and function prediction (B).

**Supplementary Figure S3.** Assembled to the chromosomal level with Hi-C data, the interaction heatmaps of *A. altus* (A), *H. odoe* (B) and *S. insignis* (C) with resolution of blocks at 500 kbp.

**Supplementary Figure S4.** The 17-mer frequency distribution of four genomes. The estimated genome size 997 M, 925 M, 793 M and 1.49 G separately.

**Supplementary Figure S5.** Comparison and annotation of mitochondrial genomes, which belong to four organisms sequenced in this study.

**Supplementary Figure S6.** The copy number of 83 genes in 69 species, which were sorted in taxonomy and phylogenetic relationships.

**Supplementary Figure S7.** The copy number of SCPP in 69 genomes. The previous 38 genes in the graph were sorted by the genome location in *L.oculatus*.
